# Visiting Professors

**Published:** 1956-10

**Authors:** 


### *Visiting Professors*

It has been said that the difference between part-time professors and whole-time professors is that the former are away from their posts for part of the time but that the latter are away the whole time. Be this as it may, it is a fact that university professors are called upon to undertake a great deal of work which is outside their terms of contract and which often takes them away for appreciable periods of time. The universities recognize this, for the work which is done in this way often helps to bring credit to the university and the contacts which are made bring distinguished visitors and may also attract junior staff and research workers of high quality. These commitments are often carried out during vacations and affect the science and medical faculties in particular. But this visiting is not confined to holders of university appointments, and Boards of Governors and Regional Hospital Boards have been generous in granting leave to distinguished consultants who have been called upon to undertake missions abroad. Apart from the local value, these visits form an important link in the ties which exist amongst the Commonwealth countries in particular and with foreign countries and thus also have a national importance. For the visitor they are always an interesting experience but in no sense can they be regarded as a holiday for the work is exacting and the distances which have to be travelled may be very great. New groups of people may be met every few days and the visitor is often strained by the hospitality and by the feeling that he has to appreciate the local circumstances and not make any statements which can be misinterpreted.

In this issue of the *Journal*, Professor Lennon gives an account of one of these visits to the Middle East. The other clinical professors in Bristol have all been called upon to undertake overseas visits during the last few years—Professor Perry to Ceylon, Professor Milnes Walker to Australia, New Zealand and Canada, and Professor Neale to the Caribbean Colonies. In addition, Mr. Eyre-Brook is just back from a tour of the colonies in the Pacific Ocean and Dr. Middlemiss is at the moment on one of his African tours.

The purpose of the visit may be to serve as a visiting teacher for a period, to act as examiner, to give one or more lectures, to hold inspections, to give advice or to collect evidence. Usually the opportunity is taken to combine a number of these functions. In the days before air travel some of these tours would have been quite impossible; flight has made some possible and made them all strenuous. They hold an important place in maintaining the British tradition, in medicine perhaps more than in any other discipline, and we are pleased to see that Bristol is taking its full share in this aspect of our national duty.

